# Modifying tumor associated macrophage function through cannabinoid receptor 2

**DOI:** 10.1186/2051-1426-3-S2-P291

**Published:** 2015-11-04

**Authors:** Taxiarhia Arabatzis, Taylor Littlefield, Jordan Watson, Jason Thole, Elizabeth McAndrew, Katherine E Hanlon

**Affiliations:** 1 University of New England, Biddeford, ME, USA

## 

In human breast cancers, tumor infiltration by tumor associated macrophages (TAMs) correlates with multiple markers of poor prognosis: higher tumor macrophage counts are associated with higher tumor grade, higher tumor vascular density, and reduced overall survival[1]. TAMs secrete biologically active molecules that promote tumor growth and metastasis[1]. Additionally, unique subpopulations of TAMs contribute to the immunosuppressive microenvironment that significantly enhances tumor genesis by limiting vital anti-tumor immune responses[2], however the mechanisms that regulate TAM development and their actions within the tumor microenvironment remain poorly understood. Pre-clinical studies demonstrate that macrophage ablation retards tumor progression[3], suggesting that TAMs may be a viable target for eliciting an anti-tumor response. We have therefore begun to analyze the effects of novel cannabinoid agonists on TAMs in a murine model of breast cancer. Macrophage expression of cannabinoid receptor 2 (CB_2_) is induced by inflammatory stimuli and regulates multiple functions including chemotaxis and antigen presentation[4]. Recent findings in our lab and by others indicate that CB_2_ agonists significantly reduce tumor burden and metastasis in several models. Although the *in vivo* anti-tumor efficacy of cannabinoids is mimicked in continuously cultured cancer cells *in vitro*, cannabinoid potency for direct elicitation of apoptosis in cancer cells is far lower, indicating that other mechanisms (perhaps other cell types within the tumor) are involved *in vivo*. We have recently obtained evidence that: 1) CB_2_ agonists mediate inhibition of primary tumor growth and metastasis *in vivo*, 2) significant infiltration of TAMs occurs within the developing tumor in this murine model, 3) administration of a CB_2_ agonist increases populations of pro-inflammatory M1 macrophages (MHC Class II-high, iNOS high) in both the primary tumor and in the spleen of tumor bearing mice, and 4) a concurrent increase in the proportion of CD8+ cytotoxic T lymphocytes is also observed within the primary tumor upon CB_2_ agonist treatment.
